# Effect of Liquid Absorbent Pads and Packaging Parameters on Drip Loss and Quality of Chicken Breast Fillets

**DOI:** 10.3390/foods10061340

**Published:** 2021-06-10

**Authors:** Marit Kvalvåg Pettersen, Julie Nilsen-Nygaard, Anlaug Ådland Hansen, Mats Carlehög, Kristian Hovde Liland

**Affiliations:** 1 Norwegian Institute of Food, Fisheries and Aquaculture (Nofima AS), Osloveien 1, 1430 Ås, Norway; post@nofima.no (J.N.-N.); Anlaug.Hansen@Nofima.no (A.Å.H.); Mats.Carlehog@Nofima.no (M.C.); 2 Faculty of Science and Technology, Norwegian University of Life Sciences (NMBU), 1432 Ås, Norway; Kristian.Liland@nmbu.no

**Keywords:** food packaging, drip loss, liquid absorbent pad, chicken breast fillet, texture, sensory evaluation

## Abstract

Visible liquid inside food packages is perceived as unattractive to consumers, and may result in food waste—a significant factor that can compromise sustainability in food value chains. However, an absorber with overdimensioned capacity may cause alterations in texture and a dryer product, which in turn may affect consumers’ satisfaction and repurchase. In this study we compared the effect of a number of liquid absorbent pads in combination with headspace gas composition (60% CO_2_/40% N_2_ and 75% O_2_/25% CO_2_) and gas-to-product volume ratio (g/p) on drip loss and quality of fresh chicken breast fillets. A significant increase in drip loss with an increasing number of liquid absorbent pads was documented. The increase was more pronounced in 60% CO_2_/40% N_2_ compared to 75% O_2_/25% CO_2_. By comparing packaging variants with a different number of liquid absorbent pads, a higher drip loss for all tested was found at g/p 1.8 compared to g/p 2.9. Total viable counts (TVC) were independent of whether there was free liquid in contact with the product, and TVC was independent of gas composition. Differentiation between the gas compositions was seen for specific bacterial analyses. While significant changes were observed using texture analysis, sensory evaluation of the chicken breast fillets did not show any negative effect in texture related attributes. This study demonstrates the importance of optimized control of meat drip loss, as product-adjusted liquid absorption may affect economy, food quality, and consumer satisfaction, as well as food waste.

## 1. Introduction

In light of the past several years’ focus on sustainability, and the global targets of the United Nations Sustainable Development goals [1] related to responsible production and consumption, the drive towards development of food systems for reducing food loss and food waste has been pronounced. Packaging in general, and especially plastics, have for the last years seen increasing public awareness of the related environmental challenges, specifically related to littering and marine debris [2]. However, one of the main functions of packaging is to protect and preserve the food in the total value chain—from the producer to the consumer. Food packaging is recognized to contribute to food waste reduction and more sustainable food value chains [1,3,4,5].

Through evaluation of the environmental impact of meat products, measured as greenhouse gas (GHG) emissions [6,7], it has been found that the packaging is only responsible for a small part of the GHG emissions [8]. Considering the small environmental footprint of the packaging compared to that of the meat products, it is clear that optimal packaging systems to avoid food loss in the value chain and food waste at consumers should be high priority, and will contribute to more sustainable food systems.

Fresh meat products contain high amounts of water. The physiological water content in muscle foods, such as chicken, beef, and pork, is approximately 75% [9]. The water-holding capacity (WHC) of the meat refers to the ability of the meat to retain its natural or added water content during postmortem processing and storage [10,11]. The high water content of meat makes these products particularly prone to microbial spoilage, making them highly perishable. Another important factor tightly linked to the high water content, and thereby also to the perceived quality of these products, is their drip loss [12]. Unavoidably, these products will exude some liquid during storage. Scientific literature in the field mainly focuses on how different aspects of prehandling can be related to excessive WHC and drip loss for red meat (pork, beef, and lamb) [13,14]. Factors like genotype, feeding, slaughtering, and chilling have been evaluated [14], as well as the effect of cutting of muscle fibers. The only packaging-related aspects studied in relation to drip loss were the effect of shrinking or non-shrinking films [13], i.e., there are relatively few studies focusing on the effect of packaging variables [12,15,16]. However, in all cases the comparison has been between MAP (80% O_2_/20% CO_2_ or 30% CO_2_/70% N_2_) and vacuum [12,16]. Payne et al. (1998) compared vacuum with CO_2_ flushing and/or the use of active packaging as an oxygen scavenger [15]. The studied storage temperatures have also varied a great deal, but no studies included storage at traditional/recommended storage temperatures at retailers in Europe [12,15,16]. Thus, to our knowledge, the effects of different MA gas compositions or gas-to-product volume ratios in relation to drip loss have not been reported for chicken meat.

A liquid absorber is often used in packages with fresh meat and fish to improve the appearance of the product. The capacity of these liquid absorbers is typically chosen by the food producers to ensure absorption of all drip loss, and may not be specifically designed for each product. An absorber with an overdimensioned capacity may cause an unnecessarily high drip loss. This can cause a dryer product and an alteration in texture. Furthermore, visible liquid in the packages can be perceived as unattractive by the consumer [17]. Sensory quality attributes such as juiciness and tenderness of the meat may be reduced, and these are important in terms of how the product is perceived by the consumer at the time of consumption [17].

There is an economic aspect to striving to limit the drip loss of muscle foods. Firstly, free liquid inside packages may be perceived as unattractive by the consumer, and may result in reduced sales [17,18]. In addition to this, a common perception seems to be that excess liquid inside packages can give rise to increased microbial growth and reduced meat quality. However, this has been disproven in a previous publication, where no such relation could be documented [18]. On the contrary, the study showed that the most attractive growth medium for bacteria is the product itself, not its exudate. Finally, the liquid lost implies a reduction in product weight and a reduced product yield for food producers. 

Another aspect in the context of drip loss is the fact that the EU regulates the use of absorbers containing superabsorbent polymers (SAP), which are considered to be active packaging devices. For non-sealed absorbers it is mandatory for food producers to use absorbers of adequate capacity in order to ensure that the absorber can absorb all liquid lost from the product. This is to ensure that there is no leakage of SAP that can come into contact with the product (Commission Regulation (EC) No 450/2009 on active and intelligent materials and articles intended to come into contact with food) [19]. Due to this, food producers may be prone to choose an absorber that has an overdimensioned capacity for the product, in order to make sure that there is no free liquid inside the packages.

A high amount of CO_2_ inside modified atmosphere packaging (MAP) is often associated with increased drip loss of the product, as CO_2_ dissolving into the product causes the WHC to decrease [20,21,22]. One of the assumed mechanisms at play is the reduction of pH in the presence of CO_2_ [23]. Others have reported negative correlation between CO_2_ content and drip loss of meat [24]. However, often overlooked is the more pronounced effect of underpressure formation inside the packages at high CO_2_ levels. CO_2_ absorbed by the product may cause package deformation and a physical squeeze on the product, resulting in increased drip loss [25,26].

In this study we wanted to systematically investigate which packaging parameters are the most determinant for influencing the drip loss—and consequently, the physicochemical, microbiological, and sensory quality—of chicken breast fillets. The initial experimental setup was designed to address the following research questions: How does the number of liquid absorbent pads affect the drip loss and quality of the meat? How is the drip loss affected by different gas atmosphere compositions in MAP? Chicken breast fillets were chosen as the model product due to their relatively high drip loss and well-known challenges related to rapid microbiological spoilage. The number of liquid absorbent pads and different gas compositions were the variables included in the main experiment. Based on the results from the first experimental setup, a follow-up experiment was designed, aimed at addressing the new emerging research question: How is the drip loss affected in MAP with CO_2_ at different gas-to-product volume (g/p) ratios? In this experiment the number of liquid absorbent pads and the gas-to-product (g/p) volume ratio were the main variables. 

## 2. Materials and Methods

### 2.1. Sample Preparation and Storage Conditions

Chicken breast fillets (breast fillet tenderloin; pectoralis major) were obtained from a local producer using a fast and highly automated process with low temperature during the process. The slaughtering was performed in the morning with continuous cooling and cutting. The approximate time for slaughtering and cooling was 3 h, followed by cutting and packaging within less than 30 min. The fillets were wrapped in plastic bags and transported chilled in distribution boxes containing approximately 10 kg each. The packaging was performed at the research institute shortly after reception and within 48 h after slaughtering. The fillets were randomly selected from the distribution boxes. Two fillets with a total average weight of 339 g (339.1 ± 4.4 g) (329.9–350.9 g) were packaged in each tray. The samples were stored in dark conditions at 4 °C.

### 2.2. Packaging Materials

The chicken breast fillets were packaged in thermoformed trays with a base web consisting of amorphous polyethylene terephthalate/polyethylene (APET/PE) (Multipet 550 μm, Wipak, Nastola, Finland). Biaxer 65 XX HFP AFM consisting of polyethylene terephthalate/polyethylene/ethylene vinyl alcohol/polyethylene (PET/PE/EVOH/PE) (Wipak (Nastola, Finland)) was applied as the top web. 

The oxygen transmission rates (OTR) of the materials were, according to the producer: 10 cm^3^/(m^2^ d) at 23 °C, 50% RH for the base web, and 5 cm^3^/(m^2^ d) at 23 °C, 50% RH for the top web.

The trays were thermoformed using a Multivac R145 thermoforming machine (Multivac, Wolfertschwenden, Germany). 

In both experiments, different numbers of liquid absorbent pads were used—0, 1, and 2 (Absorber type 109642, MP-2501 75 × 115 mm black, Færch, Denmark)—and thereby 3 different possibilities of liquid absorption for each gas composition.

### 2.3. Packaging Methods and Experimental Design

The studies encompass two experiments with chicken breast fillets. In the first and main experiment (all analyses included) (hereafter referred to as Experiment 1), the gas composition and the number for liquid absorbent pads were the experimental design factors. In this experiment, the chicken breast fillets were stored in a modified atmosphere of 60% CO_2_/40% N_2_ or 25% CO_2_/75% O_2_, and a gas-to-product volume (g/p) ratio of 1.8 was applied for all samples. Four replicates of each sample type were prepared for each sampling time, performed after 0, 6, 14, and 20 days of storage.

In the second experiment (Experiment 2), the effect of the g/p ratio and the number of liquid absorbent pads was investigated. The chicken breast fillets were stored in 60% CO_2_/40% N_2_, and two different tray sizes were used. Trays with a volume of 860 mL resulted in an initial gas-to product-volume ratio (g/p ratio) of approximately 1.8, while trays with a volume of 1390 mL gave an initial g/p ratio of approximately 2.9. Four replicates of each sample type were prepared for each sampling time, performed after 0, 7, 14, and 20 days of storage.

### 2.4. Analyses 

#### 2.4.1. Headspace Gas Analyses and Drip Loss

The headspace atmosphere of the MA packages was analyzed for CO_2_ and O_2_ levels (%) immediately after packaging and at each sampling time using a CheckMate 9900 O_2_/CO_2_ analyzer (PBI Dansensor, Ringsted, Denmark). Gas was removed from the packaging for analysis using a needle through self-sealing patches on the packages.

Drip loss was determined by initially weighing the meat, the package, and the absorbent pad(s), and calculating the increase in weight of the packages (including the absorbent pads) at each sampling. Results are given as the percentage (%) of initial muscle weight, and refer to the corresponding drip loss from the meat. These analyses were performed in all experiments. 

#### 2.4.2. Texture Analyses and Dry Matter Content

Warner–Bratzler shear force (WBSF) and dry matter content measurements were performed (in Experiment 1) for chicken breast fillets cooked after storage in different packaging conditions after 0, 6, 14, and 21 days of storage. The fillets were vacuum packed in PA/PE (70 µm) (Maskegruppen, Norway) bags and heat treated in a water bath at 70 °C for 50 min before being cooled in ice water for 50 min. The samples were stored in the vacuum bags at 4 °C until the next day. Prior to WBSF measurements, the temperature of the samples was equilibrated at 20 °C for 1 hour. The fillets were cut into rectangular pieces of 1 × 1 × 2 cm along the fiber direction. The samples were sheared perpendicularly to the fiber direction with a triangular device attached to an Instron Materials Testing Machine (model 4202, Instron Engineering Co., High Wycombe, UK). The average maximum force (given as N/cm^2^) was obtained from measurement of 6 replicates.

For determination of dry matter content, the samples were macerated/homogenized, and approximately 6 ± 0.5 g of the mass was accurately weighed into Petri dishes and oven dried at 105 °C for 18 h. The samples were weighed after drying, and the weight loss during drying was equal to the water content of the samples. The dry matter content was calculated as the percentage of the initial weight minus the water content (Dry matter content (%) = w_sample_ (%) − w_water_ (%)). Two replicates per sample variant were measured at each sampling time.

#### 2.4.3. Microbiological Analyses

The selected microbiological analyses for chicken breast fillets (Experiments 1 and 2) were total viable count (TVC), *Enterobacteriaceae,* lactic acid bacteria (LAB), and *Brochothrix thermosphacta*, performed at the time of packaging and after the selected sampling time. 

Samples of 3 × 3 cm^2^ and 1 cm depth were cut with a sterile scalpel from the surface of the meat, weighed, macerated, and diluted 1:10 with peptone water and spread using a Whitley Automated Spiral Plater (WASP) (Don Whitley Scientific Ltd., West Yorkshire, UK). In addition, in Experiment 1, after 14 days of storage, samples from the bottom surfaces of the fillets were cut and included for analyses. Appropriate 10-fold dilutions were spread in duplicate on PCA (plate count agar; Difco, Difco Laboratories, Detroit, MI, USA) for total viable counts (TVC) (incubation temperature 30 °C, 72 h, anaerobic incubation), and on MRS agar (Man, Sharpe and Rogosa agar, MRS; Oxoid, Unipath Ltd., Basingstoke, Hampshire, UK) for lactic acid bacteria (incubated at 20 °C, 48 h, anaerobic incubation). *Enterobacteriaceae* were analyzed by use of VRBGA (Violet Red Bile Glucose Agar, Oxoid, Hampshire, UK) (37 °C, 24 h, semi-aerobic conditions, cells embedded in agar with sterile overlay). *Brochothrix thermosphacta* was detected by use of STAA agar (streptomycin thallous acetate actidione) and an agar base (CM 0881 with selective supplement SR 0151E, Oxoid, Hampshire, UK) (25 °C for 48 h, aerobic incubation). Microbial counts are expressed as colony-forming units (cfu) per g. 

#### 2.4.4. Sensory Analysis 

Sensory analysis was performed on both raw and heat-treated chicken breast fillets after 14 days of storage in both modified atmospheres—25% CO_2_/75% O_2_, and 60% CO_2_/40% O_2_—with 0, 1, and 2 liquid absorbent pads (g/p 1.8) (Experiment 1). A highly trained panel of 10 assessors (10 women; aged, 37–64 years) at Nofima (Ås, Norway) performed a sensory descriptive analysis (DA) according to the “generic descriptive analysis” [27] and ISO standard 13299 [28]. The assessors are regularly tested and trained according to ISO standard 8586, and the sensory laboratory follows the practice of ISO standard 8589 [29,30]. 

In a pretest session before the main test, the assessors were calibrated on samples that were considered the most different on the selected attributes typical for raw and heat-treated chicken fillets. The results from the pretest were evaluated and discussed by the panel leader and the assessors. This calibration procedure was performed in order to arrive at a common understanding and agreement of the selected attributes. This is common practice, with the purpose being to ensure that the assessors have a common understanding of how to evaluate and rank the different sensory attributes, and to obtain consensus for each attribute among the assessors. For raw evaluation the assessors agreed upon six sensory attributes describing odor: sourness odor, metallic odor, cloying odor, sulfurous odor, fermented odor, and chlorine odor. For heat-treated evaluation, the assessors agreed upon eight sensory attributes describing flavor and texture: sourness flavor, metallic flavor, cloying flavor, sulfurous flavor, fermented flavor, hardness, juiciness, and tenderness.

The sensory evaluation of raw samples was performed on chicken fillets stored in the original packaging. The samples were first heated to room temperature. Immediately before evaluation, an opening (4 × 4 cm) was cut into the top web of the packages, and the assessors smelled the sample trays. 

For heat-treated sensory evaluation, each assessor was served one piece of chicken in a triangular shape and served from the same position of the fillet throughout the whole test. Heat treatment of chicken fillets was performed in a combi oven (Electrolux Air-o-steam, Model AOS061EANQ) at +100 °C with 100% heat for 20 min (core temperature of 72 °C). Samples were served in preheated porcelain bowls with warm metal lids and placed on a heating plate in the sensory booth. All samples were served to the panel coded with a three-digit number in duplicate following a randomized block design. 

All attributes were evaluated on an unstructured 15 cm line scale with labeled end points ranging from “no intensity” (1) to “high intensity” (9). Each assessor evaluated all samples at individual speed on a computer system for direct recording of data (EyeQuestion, Software Logic8 BV, Utrecht, The Netherlands). Tap water and unsalted crackers were available for palate cleansing.

#### 2.4.5. Statistical Analyses 

The experiments were prepared using balanced experimental designs for easy analysis of the packaging choices under investigation. Subsequent data analysis was performed using analysis of variance (ANOVA) with type II sums of squares and proportions of explained variance. Tukey’s pairwise comparisons were used to generate compact letter displays (CLDs), indicating which factor levels—e.g., different MAPs or number of absorbent pads—were not significantly different. All tests were performed using a level of significance of 0.05. The software used in the analyses was R version 4.0.4 [31] and the R package “mixlm” version 1.2.4 [32].

For sensory performance, ANOVA was conducted on the descriptive sensory data in order to identify the sensory attributes that discriminated among samples. A two-way mixed model was fitted for each of the sensory attributes, with the assessor and interaction effects considered to be random and the samples as a fixed effect. Least significance differences were calculated using Tukey’s test (*p* < 0.05). The statistical software used for the sensory analysis was EyeOpenR (Logic8 BV).

## 3. Results and Discussion

### 3.1. Effect of Liquid Absorbent Pads and Gas Composition 

**Figure 1:**
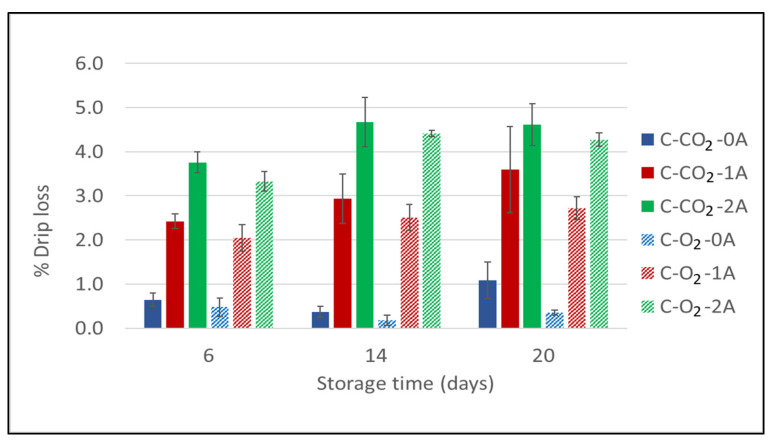
Drip loss for chicken breast fillets expressed as a percentage (%) of initial product weight as a function of storage time for chicken breast fillets (C) packaged in modified atmospheres of 60% CO_2_/40% N_2_ (C-CO_2_) and 75% O_2_/25% CO_2_ (C-O_2_) with 0, 1, or 2 liquid absorbent pads (0A, 1A, 2A). g/p ratio for all samples was 1.8 (Experiment 1). Sampling times were after 6, 14, and 20 days of cold storage. For each bar, the error bars indicate +/− one standard error.

The percentage drip loss was measured for chicken breast fillets packaged in modified atmospheres of 60% CO_2_/40% N_2_ and 75% O_2_/25% CO_2_ with 0, 1, and 2 liquid absorbent pads (Experiment 1) at selected sampling times during storage: 6 days, 14 days, and 20 days. The results presented in Figure 1 show that there is a clear positive relation regarding the number of liquid absorbent pads (i.e., increased absorbing surface) present in the packages. This implies that when increasing the number of the absorbent pads, the liquid lost from the product will increase as the absorbers draw excess liquid from the product. The tendency is evident for both packaging atmospheres (Figure 1). The measurements show a small increase in drip loss over the time of storage that is documented through these sampling times (6, 14, and 20 days) for all six sample variants. However, the most pronounced differences among the variants are already present at the first sampling time (day 6), revealing that the main part of the drip loss actually occurs during the initial days of storage. The relationship between the sample variants does not change after this. 

The gas composition and storage time had significant effects on drip loss (1.8% and 3%, respectively); however, the number of absorbent pads had the most effect (88.7%) (Appendix A Table A1). Furthermore, the measured drip loss was lower for the samples packaged in 75% O_2_/25% CO_2_ than for those packaged in 60% CO_2_/40% N_2_, though not different enough to be significant. These effects were present at all sampling times (though more pronounced towards the end of storage) and for different numbers of absorbent pads. This can be explained by the solubility of CO_2_ into the product, causing underpressure formation and a physical pressure on the product, resulting in increased drip loss [25,26]. The magnitude of the underpressure formed is proportional to the amount (percentage) of CO_2_ present in the package, while being disproportional to the g/p ratio of the packaging concept (the available volume of the gas in relation to the volume of the product). On the other hand, increasing the CO_2_ level may be beneficial in terms of improved microbiological quality—an aspect that will be considered in the following section.

**Figure 2:**
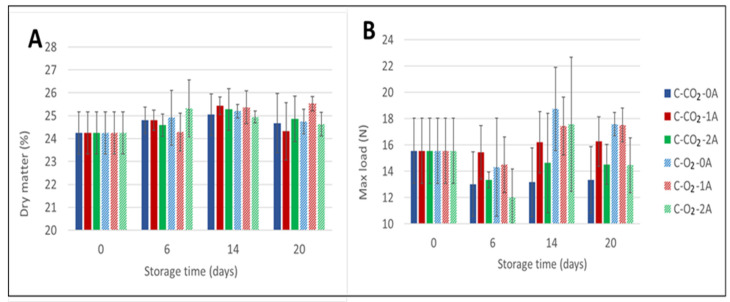
Percentage dry matter content measured for chicken breast fillets (**A**) (two replicates per sample variant) and Warner–Bratzler maximum shear force (N/cm^2^) (WBSF) required to cut cooked samples of chicken breast fillets (**B**) (presented as an average of 6 replicates per sample variant). The chicken breast fillets (C) were stored in modified atmospheres of 60% CO_2_/40% N_2_ (C-CO_2_) and 75% O_2_/25% CO_2_ (C-O_2_) (g/p 1.8) with 0, 1, or 2 liquid absorbent pads (0A, 1A, 2A) (Experiment 1) and sampling times of 0, 6, 14, and 20 days.

In accordance with the observed increase in drip loss during storage, the dry matter content of the chicken breast fillets also increased during the initial part of storage (from day 0 to day 6), to varying extents, for all sample variants (Figure 2A). For fillets stored in a CO_2_-rich atmosphere, the dry matter content of the chicken meat increased until 14 days of storage, followed by a decrease in dry matter content. For samples stored in an O_2_-rich atmosphere the effect of storage time on the dry matter content was more ambiguous, and no clear correlation could be deducted from the results.

In general, for all samples there was a tendency for the dry matter content to increase from the time of packaging to the end of storage, but the dry matter content varied among the sample variants and by the time of storage. Regarding the number of liquid absorbent pads, no clear correlation between an increased number of pads and dry matter content could be documented—neither for the samples in CO_2_-rich atmospheres, nor for those in O_2_-rich atmospheres. Correlation analyses showed that the correlation between dry matter and drip loss was −0.166 in CO_2_-rich atmospheres, while no correlation (−0.001) was observed in O_2_-rich atmospheres. Furthermore, the number of pads had no significant effect as a main factor (Appendix A Table A1).

The effects of the number of liquid absorbent pads and of gas composition were also evaluated through texture analysis using the Warner–Bratzler method. As displayed in Figure 2B, for fillets stored in CO_2_-rich atmospheres the measured WBSF shows a net decrease from the time of packaging (15.6 N/cm^2^) to the end of storage for samples stored without absorbent pads (0A) (13.4 N/cm^2^) and for those stored with two absorbent pads (2A) (14.5 N/cm^2^). For fillets stored with one absorbent pad (1A) the measured WBSF was practically unchanged throughout storage. In general, the measured differences between 0, 1, and 2 absorbent pads for this gas composition were very small. In addition, according to one-way ANOVA—computed by defining a six-level factor with the combinations of gas composition and number of liquid absorbent pads—there were no significant differences between these samples at any storage time (Appendix A Table A2). Although all main factors (gas composition, number of absorbent pads, and storage time) were significant (Appendix A Table A1), the variance explained by these factors was relatively low (7.2%, 6.6%, and 13.2%, respectively), leaving 59% as unexplained and a corresponding R^2^ of 41%, i.e., other sources of variation dominated the WBSF.

For storage in high O_2_-atmospheres, an initial reduction in maximum force was measured for all three numbers of absorbent pads (0, 1, or 2 absorbent pads) added. Perhaps most interesting in these results is the increase in measured maximum force between day 6 and day 14 of storage, indicating a decrease in tenderness of the fillets cooked after this time of storage, seen only for high-O_2_ samples. The measured shear force dropped at the last sampling day (day 20) of storage regardless of the number of absorbent pads. For fillets stored zero (0A) or with one absorbent pad (1A), the texture evaluated by WBSF was measured to be higher (17.2 N/cm^2^ and 17.5 N/cm^2^) at the end of storage compared to the initial level (15.6 N/cm^2^), indicating a decrease in meat tenderness towards the end of storage. For chicken stored with two absorbent pads (2A), the measured WBSF was reduced at the end of storage (14.5 N/cm^2^) compared to the start (15.6 N/cm^2^). However, according to one-way ANOVA, no significant differences between the samples stored in high-O_2_ atmospheres were detected at any sampling time. The only significant differences were observed between samples stored in different atmospheres after 20 days of storage (Appendix A Table A2). The physicochemical origin of the observed differences between samples stored in high-CO_2_ and high-O_2_ atmospheres is unknown. For differences in tenderness between chicken fillets to be of importance for the perceived quality, the numeric differences in measured shear force would assumedly need to be a great deal larger. Again, even though the fillet selection was randomized at packaging, some of the measured variations may be a result of individual variations.

**Figure 3:**
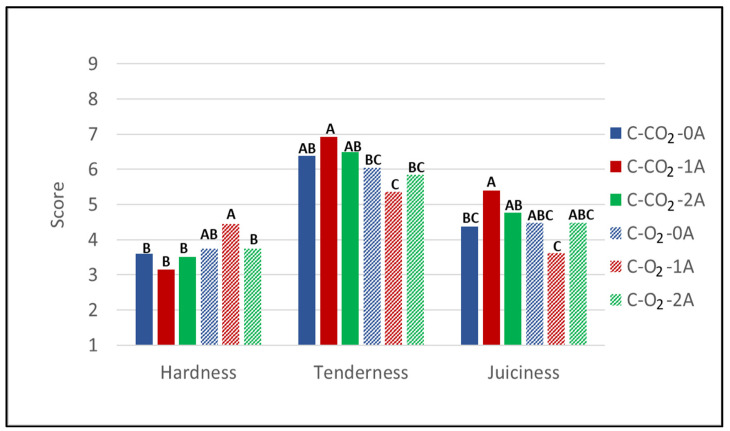
Sensory intensity scores (scale 1–9) for the textural attributes hardness, tenderness, and juiciness of heat-treated chicken breast fillets (C) stored for 14 days in modified atmospheres of 60% CO_2_/40% N_2_ (C-CO_2_) and 75% O_2_/25% CO_2_ (C-O_2_) with 0, 1, or 2 liquid absorbent pads (0A, 1A, 2A). g/p ratio for all samples was 1.8 (Experiment 1).

The results from sensory evaluation of the textural traits hardness, tenderness, and juiciness of heat-treated chicken breast fillets (Figure 3) reveal that there are significant differences in the perceived texture of the meat as affected by the number of absorbent pads (flavor evaluation of heat-treated chicken breast fillets and odor evaluation of raw chicken are presented in Appendix A Table A3, and will be discussed in relation to microbial growth).

However, the trend is not systematic across gas compositions, as it does not increase/decrease with the increasing number of pads independent of the gas composition, i.e., the only significant difference is found in the interaction between the number of absorbent pads and the packaging gas. A significantly higher score of hardness with one absorbent pad (1A) compared to two absorbent pads (2A) was observed for samples stored in high-O_2_ atmospheres, while regarding the juiciness of chicken stored in high-CO_2_ atmospheres, a lower score for samples stored without absorbent pads (0A) compared to one absorbent pad (1A) was observed. In this experiment, tenderness and juiciness both correlated positively with dry matter content (0.231 and 0.210, respectively) and negatively with WBSF (−0.517 and −0.197, respectively), while hardness did not correlate with dry matter (−0.040) but correlated positively with WBSF (0.397). However, although there were significant differences in sensory scores among different numbers of absorbent pads, the differences were less than 2 in score (hardness 3.75 and 4.45; juiciness 4.37 and 5.40), i.e., less than what is presumed observable by consumers. In addition, no significant differences in dry content or texture measured as maximum force (WBSF) (neither number of absorbent pads nor gas composition) were observed for the samples stored for 14 days. This implies that for this product consumers will not be able to identify differences in the textural quality of fillets packaged with liquid absorbent pads overdimensioned for the product’s drip loss.

With regard to the effect of modified atmosphere composition on the textural attributes, the samples stored in high-O_2_ atmospheres were evaluated with slightly higher sensory intensity scores for hardness than the ones stored in high-CO_2_ atmospheres. This is supported by the fact that the sample variants stored in high-O_2_ atmospheres were evaluated to have somewhat lower intensity scores on tenderness than the sample variants stored in high-CO_2_ atmospheres. For juiciness—an important quality trait for chicken breast fillets—no clear tendency can be observed when comparing the sample variants stored in high-O_2_ compared to high-CO_2_ modified atmospheres. However, the gas composition significantly affected the texture in samples with one absorbent pad (1A), as shown by lower scores of tenderness and juiciness and higher scores of hardness in the samples stored in high-O_2_ atmospheres compared to high-CO_2_ atmospheres. This finding is in accordance with the results of the Warner–Bratzler shear force measurements, which, as discussed, displayed the largest relative differences between the two gas compositions at measurement after 14 days of storage—the same storage time at which sensory evaluation was performed. Still, the differences in sensory scores are small and will most likely not be detectable by the average consumer. Geesink et al. (2015) stated that the effect of high oxygen on tenderness of meat has been reported in a number of studies [33]. To the best of our knowledge, reported effects of packaging atmosphere on textural attributes of chicken/poultry meat are rather scarce. Rossaint et al. (2015) reported no significant difference in texture (measured as sensory attribute) for poultry stored in a high-oxygen atmosphere (70% O_2_/30% CO_2_) compared to 70% N_2_/30% CO_2_ nitrogen [34].

Regarding texture measured as maximum force (WBSF), a majority of publications have been on beef and pork meat, and often conducted comparisons of high-oxygen atmospheres with vacuum (oxygen- and CO_2_-free atmosphere) [35,36,37,38]. Lagersted et al. (2011) reported that storage in MAP with high oxygen resulted in higher shear force and negatively affected the juiciness and tenderness of beef steaks compared to vacuum [37]. Moczkowska et al. (2017) reported a decrease in shear force (WBSF) for beef stored in an oxygen-free atmosphere (vacuum), while increased WBSF was observed when stored in a high-oxygen atmosphere [36]. Similarly, Zakrys-Waliwander et al. (2012) detected significantly lower WBSF in beef steaks stored in vacuum compared to a high-O_2_ atmosphere after 8 and 14 days, but not after 4 days of storage [38]. For pork meat (porcine longissimus dorsi) a decrease in tenderness was detected already after 4 days of storage in a high-oxygen atmosphere, with further decrease until 14 days of storage, compared to an increase in tenderness when stored in an oxygen-free atmosphere (vacuum) [39]. In oxygen-rich atmospheres the potential for increased oxidation is present, and oxidation of protein can influence properties such as water-holding capacity, and lead to changes in texture, such as tenderness [36,39].

The TVC at the start of storage (day 0) was approximately 2.6 log cfu/g for all sample variants (Appendix A Table A4). In general, the increase in TVC developed in a parallel manner for sample variants in high-O_2_ and high-CO_2_ atmospheres, with a slightly lower bacterial growth on the samples in high-CO_2_ atmospheres, reaching 6.7–7.1 log cfu/g after 20 days of storage for the high-CO_2_ variants, and 7.3–7.5 log cfu/g for the high-O_2_ atmosphere samples. Overall, there was a small but significant effect of gas composition on TVC (Appendix A Table A4). Previous results show similar total viable count numbers for products packaged with and without high levels of oxygen (though with low CO_2_-levels; 30% CO_2_/70% N_2_ and 30% CO_2_/70% O_2_) [40], whereas in the present study high levels of CO_2_ resulted in lower TVCs compared to high-O_2_ atmospheres. Statistical analysis also confirmed that gas composition had a significant effect on the TVC (although only 1.7% explained variance), while there was no significant effect of different numbers of absorbent pads on TVC. Hence, the microbiological growth is independent of whether or not there is free liquid in the packages.

This finding was further supported by results from additional analysis performed at day 14 of storage by sampling from the bottom surfaces of the fillets that were in contact with available visible liquid and comparing the TVCs measured in these samples to the TVCs from the top surfaces of the fillets at the same sampling day (Appendix A Table A4). The TVC data for the top and bottom samples for all six packaging variants are summarized in Appendix A Table A4. For samples stored in high-CO_2_ atmospheres, a significantly lower bacterial level was measured for samples taken from the bottom surface (and in contact with visible liquid if present) compared to samples from the top surface of the fillets (with two absorbent pads (2A)). However, no such differences in bacterial growth for samples stored in high-O_2_ atmospheres with different numbers of absorbent pads could be detected. Hence, free liquid inside packages does not give rise to increased microbiological activity. Even in the packaging variants without an absorbent pad present (0A), the microbiological growth at the bottom and top of the fillets was similar—not significantly different—for the same packaging conditions, as well as similar to the measured TVC levels for packaging with the highest number of absorbent pads (no free liquid inside the packages). This is a significant finding, as it disproves the common perception that packaged meat with excess liquid inside the packages has a poorer microbial quality [18]. Dissemination of these results to the food industry, retailers, and consumers could contribute to reducing food waste in this product category.

Regardless of the number of absorbent pads added, growth of *Brochothrix thermosphacta* reaches a level of approximately 7 log cfu/g for fillets in high-O_2_ atmospheres and 3–4 log cfu/g in high-CO_2_ atmospheres after 20 days of storage (Appendix A Table A4). This is as expected for this bacterium; *Brochothrix* grows fast in the presence of O_2_, and it is also able to adapt to an anaerobic environment, though then at a much slower growth rate, which is in accordance with previous studies [34,41].

Lactic acid bacteria increased from 2.45 log cfu/g to approximately 7 log cfu/g (7.12–7.31 log cfu/g and 6.67–7.22 log cfu/g in high-CO_2_ and high-O_2_ atmospheres, respectively) over a 20-day storage period. Levels of *Enterobacteriaceae* were measured to be about 2 log cfu/g until 15 days of storage, followed by an increase to approximately 3 log cfu/g in high-O_2_ atmospheres (2.23–3.17 log cfu/g), and slightly higher—approximately 4 log cfu/g—in high-CO_2_ atmospheres (4.02–4.27 log cfu/g) after 20 days of storage. According to analyses of variance, the number of liquid absorbent pads had no significant effect on the levels of lactic acid bacteria and *Enterobacteriaceae.* No significant differences were observed at any sampling time within each packaging gas (Appendix A Table A4), and the variance was mainly explained by the storage time (Appendix A Table A1). Moreover, no significant effect of the number of absorbent pads in the packages on the growth of *B. thermosphacta* could be seen in these results, but in this case most of the variance was explained by gas composition and storage time (45.4% and 42%, respectively).

Sensory evaluation of the odor of the raw chicken showed similar trends as the bacterial growth, with no significant effect of different numbers of liquid absorbent pads but clear effect of the gas composition (Appendix A Table A3). In all evaluated attributes, significant differences were detected, with slightly higher scores for sourness and metallic odors and slightly lower scores for chlorine odor in chicken stored in high-CO_2_ atmospheres compared to high-O_2_ atmospheres. However, for the other attributes, the differences were more pronounced. Chicken stored in high-O_2_ atmospheres had high scores (6.88–7.28) for fermented and cloying (6.48–6.89) odors compared to storage in high-CO_2_ atmospheres (2.14–2.87 and 3.37–3.94, respectively). The opposite was the case regarding the sulfurous odor: 1.97–2.07 in high-O_2_ atmospheres and 5.38–6.23 in high-CO_2_ atmospheres. For heat-treated chicken the only significant flavor attributes were sourness and fermented flavors, though the scores were relatively low (below 3.37) and the difference was less than 2 units.

### 3.2. Effect of Liquid Absorbent Pads and G/P Ratio

The g/p ratio applied in the first experiment in the study (g/p 1.8) is comparable to what is commonly used for modified atmosphere packaging on the market in Norway. Based on the findings of this first and main part of the study, it was relevant to look into the effect of varying the g/p ratio, i.e., the amount of carbon dioxide gas available to the product, and how it affects the drip loss and microbiological quality of the chicken breast fillets when packaged with 0, 1, or 2 liquid absorbent pads. Therefore, the second part of the study includes both g/p 1.8 (as for Experiment 1) and a higher g/p ratio of 2.9.

**Figure 4:**
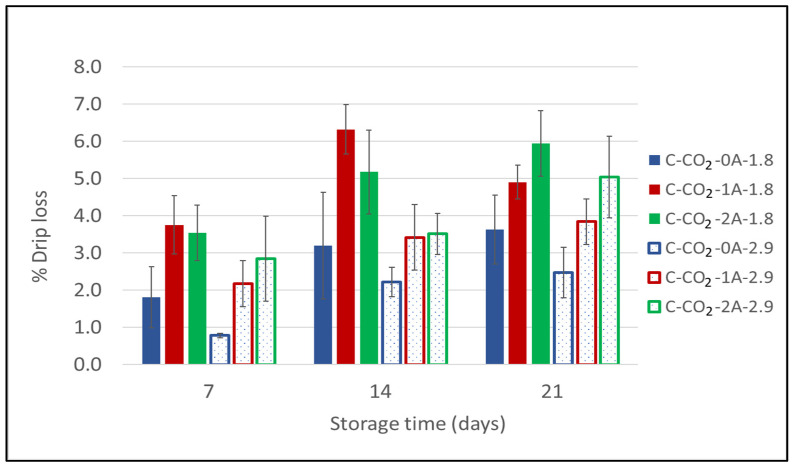
Drip loss for chicken breast fillets expressed as a percentage (%) of initial product weight as a function of storage time for chicken breast fillets (C) packaged in modified atmospheres of 60% CO_2_/40% N_2_ (C-CO_2_) with 0, 1, or 2 liquid absorbent pads (0A, 1A, 2A) and two different g/p ratios (1.8 and 2.9) (Experiment 2). Sampling times were after 7, 14, and 20 days of cold storage.

Carbon dioxide has a well-documented antimicrobial effect [13,42,43,44]. According to Renerre et al. (1990), Dalgaard et al. (1993), and Randell et al. (1999), the drip loss increases with increased CO_2_ content in the packages [20,21,22]. However, Holck et al. (2014) showed that drip loss is not solely dependent on the amount of CO_2_, with higher drip loss in chicken packed in 100% CO_2_ compared to 100% CO_2_ with the addition of a CO_2_ emitter [25]. Thus, underpressure formation with high amounts of CO_2_ in rigid packages may result in an excessive drip loss from the product, due to physical pressure as the package is compressed [26]. Figure 4 displays the drip loss results from the second experiment on chicken breast fillets. Firstly, this follow-up experiment confirms the results obtained in the initial experiment: the drip loss increases when increasing the number of liquid absorbent pads. After 20 days of storage, two absorbent pads (2A) resulted in significantly higher drip loss compared to without absorbent pads (0A). However, after 7 and 14 days of storage, significantly higher drip loss was detected in packages with one absorbent pad (1A) compared to those without absorbent pads (0A) at g/p 1.8 (Appendix A Table A2). Secondly, the figure shows that there is a general tendency towards higher drip loss for samples in modified atmospheres at g/p 1.8 compared to g/p 2.9. By statistical analysis, this effect was found to be significant, with higher drip loss at g/p 1.8 compared to g/p 2.9 with one absorbent pad (1A) after both 7 and 14 days of storage. Analysis of variance shows that all main factors (g/p ratio, number of absorbent pads and storage time) were significant (Appendix A Table A1), with explained variances of 16.7%, 26.1%, and 31.6%, respectively. At a higher g/p ratio (and identical gas composition) there is a larger amount of CO_2_ present in the package and, thus, a higher amount of CO_2_ that can be dissolved into the product. This implies that when CO_2_ is absorbed by the product the underpressure formed will be relatively small in the packages with g/p 2.9 compared to in the packages with g/p 1.8. The samples with g/p 2.9 consequently have a lower drip loss due to a less pronounced physical pressure on the chicken breast fillets. The variance in the measurements reflects the natural individual variations between the products.

Statistical analysis of variance showed that the main differences in TVC were explained by storage time (98.4%) (Appendix A Table A1). Some differences in TVC were detected after 14 days of storage, but there were no significant differences for samples stored for 20 days, showing similar bacterial counts as for the previous TVC results (Appendix A Table A4). This indicates that the microbial growth on the chicken breast fillets is not affected by changing the g/p ratio—and thereby, the amount of available CO_2_—for this packaging concept. Due to the antimicrobial effects of CO_2_, increasing the g/p ratio may have been expected to produce an increased antimicrobial effect of the packaging gas. However, the percentage of CO_2_ in the headspace was the same, and the results demonstrate that the amount of CO_2_ available at a g/p of 1.8 is adequate to produce a comparable effect of CO_2_ in terms of microbiological quality as measured for a g/p of 2.9. These results also support the finding that microbiological growth on the chicken fillets is independent of the presence of or the number of liquid absorbent pads in the packages, i.e., whether or not there is free liquid inside the packages.

## 4. Conclusions

A significant increase in drip loss with an increasing number of liquid absorbent pads was documented for chicken breast fillets. The percentage of dry matter content increased during storage; however, only minor effects of gas composition and the number of absorbent pads could be detected. Sensory properties related to the texture were not significantly affected by the number of pads for the fillets. Microbiological analyses showed that TVC was independent of the number of absorbent pads, i.e., whether or not there was free liquid in the packages. This is an interesting finding as it disproves the common belief that excess liquid inside packages results in a higher bacterial load on the product. By comparing two different packaging gases in MAP, it was confirmed that for both packaging gases increased numbers of liquid absorbent pads in the packages led to increased drip loss. Still, there was no pronounced effect on sensory quality (texture), though regardless of liquid absorption, higher intensity scores of negatively associated odor attributes were detected for the high-O_2_ atmospheres compared to the high-CO_2_ atmospheres.

This study demonstrates the importance of product-adjusted capacity of liquid absorbers in order to maintain product yield, which can have economic benefits for food producers and retailers. Visible free liquid inside the packages did not affect the bacterial load, but might result in rejection by the consumer due to unattractive packages, which might have an effect in terms of increased food waste.

## Data Availability

The data presented in this study are available on request from the corresponding author.

## Appendix A

**Table A1:** Summaries of analyses of variance for measurements on chicken breast fillets (Experiments 1 and 2). Numbers show percentage of variation per factor/interaction.

Chicken Breast Fillet (Experiment 1): G/P 1.8							
Factor/Effects	Drip Loss	Total Viable Counts	Lactic Acid Bacteria	*Brochothrix thermosphacta*	*Enterobacteriaceae*	Max Load	Dry Matter Content
Gas composition (G)	1.8 ***	1.7 ***	0	45.4 ***	5.2 ***	7.2 **	0.6
Storage time (T)	3.0 ***	95.7 ***	95.4 ***	42.0 ***	58.0 ***	13.2 **	6.4
Liquid absorbent pads (A)	88.7 ***	0	0.3 °	0.3	0.6	6.6 *	0.1
G × T	0.3	-	0.9 ***	4.1 ***	9.5 ***	6.3 °	-
G × A	0.1	-	0.3 *	0.8 *	0.9	6.5 *	-
T × A	2.0 ***	-	0.4 °	1.5 **	2.2	1.2	-
Residuals (Error)	4.1	2.6	2.7	5.9	23.6	59.0	92.9
R^2^	95.8	97.4	97.3	94.1	76.4	41.0	7.1
**Chicken Breast Fillet (Experiment 2): Gas Composition: 60% CO_2_/40% N_2_**							
	**Drip Loss**	**Total Viable Counts**	**Lactic Acid** **Bacteria**	***Brochothrix thermosphacta***	***Enterobacteriaceae***		
Gas volume/product volume ratio (GP)	16.7 ***	0.1 °	0.1 *	5.7 ***	8.1 ***		
Storage time (T)	26.1 ***	98.4 ***	97.3 ***	71.7 ***	66.5 ***		
Liquid absorbent pads (A)	31.6 ***	0	0.2 *	1.1 °	2.5 *		
GP × T	-	0	0	6.7 ***	0		
GP × A	-	0.2 *	0.5 ***	0.4	3.5 **		
T × A	-	0.2 °	0.9 ***	2.8 *	0.3		
Residuals (Error)	25.6	1.1	1.0	11.7	19.1		
R^2^	74.1	98.9	99.0	88.4	80.9		

*p*-values are indicated as 0 *** 0.001 ** 0.01 * 0.05 ° 0.1 e.g., one star for *p*-values between 0.01 and 0.05.

**Table A2:** One-way analyses of variance for drip loss, dry matter content and Warner–Bratzler shear force (N/cm^2^) (maximum load) for each recorded day of storage of chicken breast fillets (Experiment 1). The one-way ANOVAs were computed by defining a six-level factor with the combinations of gas composition and liquid absorbent pads. Tukey’s letters signify which of the six combinations of gas composition and liquid absorbent pad numbers are not significantly different (*p*-value > 0.05), i.e., levels sharing a letter are not significantly different. In the lower part of the table (Experiment 2), gas volume/product volume replaces gas composition.

Chicken Breast Fillet (Experiment 1): G/P 1.8						
	Gas Composition	Number of Liquid Absorbent Pads	0 Days	6 Days	14 Days	20 Days
**Drip loss**	60% CO_2_/40% N_2_	0	-	0.65 C	0.37 C	1.09 C
		1	-	2.42 B	2.93 B	3.60 AB
		2	-	3.76 A	4.67 A	4.61 A
	25% CO_2_/75% O_2_	0	-	0.48 C	0.18 C	0.35 C
		1	-	2.05 B	2.51 B	2.72 B
		2	-	3.33 A	4.41 A	4.27 A
**Dry matter Content**	60% CO_2_/40% N_2_	c	24.25	24.81 A	25.05 A	24.68 A
		1	24.25	24.80 A	25.44 A	24.32 A
		2	24.25	24.59 A	25.28 A	24.87 A
	25% CO_2_/75% O_2_	0	24.25	24.91 A	25.20 A	24.75 A
		1	24.25	24.29 A	25.37 A	25.52 A
		2	24.25	25.32 A	24.94 A	24.63 A
**Warner–Bratzler** **shear force (maximum load)**	60% CO_2_/40% N_2_	0	15.56	13.02 A	13.17 A	13.35 B
		1	15.56	15.46 A	16.21 A	16.27 AB
		2	15.56	13.35 A	14.63 A	14.53 AB
	25% CO_2_/75% O_2_	0	15.56	14.3 A	18.74 A	17.59 A
		1	15.56	14.51 A	17.44 A	17.52 A
		2	15.56	12.02 A	17.58 A	14.47 AB
**Chicken Breast Fillet (Experiment 2): Gas Composition: 60% CO_2_/40% N_2_**						
	**Gas Volume/** **Product Volume**	**Number of Liquid Absorbent Pads**	**0 Days**	**7 Days**	**14 Days**	**20 Days**
**Drip loss**	1.8	0	-	1.81 BC	3.20 BC	3.63 BC
		1	-	3.64 A	6.05 A	5.12 AB
		2	-	3.54 AB	5.17 AB	5.95 A
	2.9	0	-	0.78 C	2.22 C	2.47 C
		1	-	2.18 ABC	3.42 BC	3.84 BC
		2	-	2.84 AB	3.51 BC	5.04 AB

**Table A3:** Sensory intensity scores (scale 1–9) for flavor attributes for heat-treated samples and odor attributes for raw chicken breast fillets stored for 14 days in modified atmospheres of 60% CO_2_/40% N_2_ and 75% O_2_/25% CO_2_ with 0, 1, or 2 liquid absorbent pads (Experiment 1). Levels sharing a letter are not significantly different (*p* < 0.05) between the chicken breasts samples, as measured by two-way ANOVA and Tukey’s multiple comparison test.

Flavor of Heat-Treated Chicken—14 Days of Storage									
Packaging Variable: Gas Composition	Number of Liquid Absorbent Pads	Sourness	Metallic	Cloying	Sulfurous	Fermented	Hardness	Tenderness	Juiciness
60%CO_2_/40% N_2_	0	2.87 AB	4.23	3.88	2.89	1.71 B	3.59 B	6.38 AB	4.37 BC
	1	2.89 AB	4.19	4.08	2.61	1.89 B	3.16 B	6.92 A	5.40 A
	2	3.26 A	4.38	3.38	2.35	1.48 B	3.50 B	6.49 AB	4.77 AB
25%CO_2_/75% O_2_	0	3.13 A	3.79	3.67	2.73	2.43 AB	3.75 AB	6.04 BC	4.49 ABC
	1	1.80 B	3.56	4.55	3.25	3.37 A	4.45 A	5.35 C	4.63 C
	2	2.39 AB	3.63	4.39	2.96	2.59 AB	3.74 B	5.84 BC	4.48 ABC
***p*-value**		0.0058	0.0556	0.1608	0.2917	0.0008	0.0001	0.0000	0.0001
**Odor of Raw Chicken—14 Days of Storage**									
		**Sourness**	**Metallic**	**Cloying**		**Sulfurous**	**Fermented**	**Chlorine**	
60% CO_2_/40% N_2_	0	2.58 A	4.42 A	3.66 B		6.23 A	2.36 B	1.35 B	
	1	2.87 A	4.39 A	3.37 B		5.38 A	2.14 B	1.31 B	
	2	2.40 A	4.46 A	3.94 B		5.62 A	2.87 B	1.28 B	
25% CO_2_/75% O_2_	0	1.23 B	2.26 B	6.89 A		2.07 B	7.24 A	3.41 A	
	1	1.30 B	2.28 B	6.81 A		2.07 B	7.28 A	3.48 A	
	2	1.24 B	2.42 B	6.48 A		1.97 B	6.88 A	3.62 A	
***p*-value**		0.0000	0.0000	0.0000		0.0000	0.0000	0.0000	

**Table A4:** Microbiological content measured as total viable count of bacteria (TVC), lactic acid bacteria (LAB), *Brochothrix thermosphacta,* and *Enterobacteriaceae* given as log cfu/g for chicken breast fillets (Experiment 1 and 2) as a function of storage time, packaging variables, gas composition (60% CO_2/_40% N_2_ and 25% CO_2/_75% O_2_), or g/p ratio (1.8, 2.9, 2.0 and 2.1), with 0, 1, or 2 liquid absorbent pads. Sampling times after 6 (7), 14, and 20 days of cold storage. Values are mean ± standard deviation (*n* = 3).

Storage Time (Days)	Packaging Variable	Number of Liquid Absorbent Pads	Total Viable Counts	Lactic Acid Bacteria	*Brochothrix thermosphacta*	*Enterobacteriaceae*
Chicken Breast Fillet (Experiment 1): G/P 1.8						
	Gas Composition					
0			2.64 ± 0.26	2.45 ± 0.36	2.0 ± 0.0 **	2.64 ± 0.13 **
6	60% CO_2_/40% N_2_	0	3.04 ± 0.28 B	2.83 ± 0.39 B	1.50 ± 0.39 ** C	1.63 ± 0.41 ** A
		1	3.54 ± 0.19 AB	2.73 ± 0.25 A	1.42 ± 0.24 ** C	1.42 ± 0.24 ** A
		2	3.52 ± 0.29 AB	3.61 ± 0.32 A	1.42 ± 0.24 C	1.82 ± 0.46 A
	25% CO_2_/75% O_2_	0	3.83 ± 0.14 A	3.87 ± 0.16 A	3.48 ± 0.35 A	1.99 ± 0.11 A
		1	3.72 ± 0.17 A	3.65 ± 0.21 A	3.29 ± 0.40 AB	1.82 ± 0.45 A
		2	3.56 ± 0.30 AB	3.59 ± 0.41 A	2.22 ± 1.07 ** BC	1.68 ± 0.50 A
14	60% CO_2_/40% N_2_	0	5.59 ± 0.09 AB	5.78 ± 0.0 * A	2.50 ± 0.81 B	2.10 ± 0.98 BC
		0 (Bo)	5.65 ± 0.38 AB	5.70 ± 0.0 * AB	2.44 ± 0.45B	3.27 ± 0.16 AB
		1	5.71 ± 0.0 AB	5.70 ± 0.0 * AB	2.10 ± 0.0 B	1.96 ± 0.0 BC
		1 (Bo)	5.50 ± 0.41 B	5.60 ± 0.0 ** AB	1.68 ± 0.45B	2.07 ± 0.75 BC
		2	5.55 ± 0.30 AB	5.60 ± 0.0 * AB	2.93 ± 0.59 B	1.72 ± 0.68 BC
		2 (Bo)	4.69 ± 0.40 C	4.85 ± 0.32 C	2.04 ± 1.11B	1.0 ± 0.0 ** C
	25% CO_2_/75% O_2_	0	6.06 ± 0.15 AB	5.60 ± 0.0 * AB	5.45 ± 0.30 * A	1.83 ± 0.81 BC
		0 (Bo)	6.23 ± 0.08 A	5.78 ± 0.0 * A	5.24 ± 0.19 A	3.86 ± 0.32 A
		1	5.84 ± 0.30 AB	5.46 ± 0.17 B	5.70 ± 0.0 * A	1.94 ± 0.66 BC
		1 (Bo)	5.86 ± 0.20 AB	5.78 ± 0.0 * A	5.40 ± 0.15A	2.57 ± 1.36ABC
		2	5.96 ± 0.32 AB	5.63 ± 0.05 * AB	5.61 ± 0.07 * A	1.12 ± 0.24 C
		2 (Bo)	5.56 ± 0.29 AB	5.51 ± 0.06 * B	5.13 ± 0.09A	1.12 ± 0.34 ** C
20	60% CO_2_/40% N_2_	0	6.94 ± 0.09 BC	7.12 ± 0.14 AB	4.08 ± 0.62 B	4.27 ± 0.87 * A
		1	6.66 ± 0.33 C	7.31 ± 0.43 A	2.92 ± 0.59 C	4.13 ± 0.12 A
		2	7.07 ± 0.18 ABC	7.31 ± 0.25 A	3.99 ± 0.72 BC	4.02 ± 0.07 A
	25% CO_2_/75% O_2_	0	7.29 ± 0.22 AB	6.66 ± 0.08 B	6.86 ± 0.39 A	2.28 ± 0.96 B
		1	7.36 ± 0.21 AB	6.80 ± 0.16 AB	6.93 ± 0.15 A	3.17 ± 0.46 AB
		2	7.53 ± 0.25 A	7.22 ± 0.25 A	7.40 ± 0.36 A	2.91 ± 0.61 AB
**Chicken Breast Fillet (Experiment 2): Gas Composition: 60% CO_2_/40% N_2_**						
**Storage Time (Days)**	**Packaging Variable**	**Number of Liquid Absorbent Pads**	**Total Viable Counts**	**Lactic Acid Bacteria**	***Brochothrix thermosphacta***	***Enterobacteriaceae***
	**Gas/Product Volume Ratio**					
0			3.30 ± 0.41	2.11 ± 0.31	1.55 ± 0.24	1.30 ± 0.0
7	1.8	0	3.48 ± 0.04 ABC	3.65 ± 0.05 AB	1.30 ± 0.0 ** A	1.77 ± 0.38 A
		1	3.41 ± 0.12 BC	3.41 ± 0.17 AB	1.30 ± 0.0 ** A	1.89 ± 0.68 ** A
		2	3.80 ± 0.29 A	3.74 ± 0.28 A	1.38 ± 0.15 A	2.10 ± 0.43 A
	2.9	0	3.53 ± 0.09 ABC	3.49 ± 0.08 AB	1.45 ± 0.30 ** A	1.30 ± 0.0 ** A
		1	3.82 ± 0.16 AB	3.68 ± 0.12 AB	1.30 ± 0.0 ** A	1.50 ± 0.24 ** A
		2	3.29 ± 0.12 C	3.32 ± 0.13 B	1.30 ± 0.0 ** A	1.30 ± 0.0 ** A
14	1.8	0	6.22 ± 0.1 B	6.30 ± 0.0 *	2.18 ± 0.67 C	3.95 ± 0.13 A
		1	6.42 ± 0.10 A	6.30 ± 0.0 *	2.72 ± 0.57 BC	3.94 ± 0.33 A
		2	6.43 ± 0.04 A	6.30± 0.0 *	2.30 ± 0.61 C	3.75 ± 0.45 AB
	2.9	0	6.43 ± 0.02 A	6.54± 0.0 *	3.20 ± 1.12ABC	2.45 ± 1.10 B
		1	6.40 ± 0.01 A	6.54± 0.0 *	4.57 ± 0.66 A	3.37 ± 0.59 AB
		2	6.00 ± 0.0 * C	5.48± 0.0	4.02 ± 0.04 AB	3.72 ± 0.35 AB
20	1.8	0	7.34 ± 0.16 A	7.29 ± 0.11 AB	3.68 ± 0.29 A	4.22 ± 0.34 A
		1	7.35 ± 0.15 A	7.20 ± 0.10 AB	3.09 ± 0.79 A	4.03 ± 0.63 A
		2	7.40 ± 0.19 A	7.38 ± 0.11 A	4.34 ± 0.35 A	3.71 ± 0.47 AB
	2.9	0	7.13 ± 0.16 A	7.00 ± 0.11 B	3.94 ± 0.48 A	2.69 ± 0.81 B
		1	7.20 ± 0.16 A	7.27 ± 0.09 AB	4.00 ± 0.0 ** A	3.48 ± 0.68 AB
		2	7.53 ± 0.33 A	7.41 ± 0.23 A	4.36 ± 0.85 A	4.11 ± 0.61 A

(Bo) = sampling from the bottom surfaces of the chicken breast fillets. (*) value given as > (too low dilutions applied—at least two replicates over growth and not possible to count); (**) value given as < (too high dilutions applied–at least two replicates below detection limit at this dilution). Means sharing letters (Tukey) within the same column and storage time are not significantly different (*p* > 0.05) (one-way ANOVA with six levels per day).
